# The Absence of Association Between NQO1 rs1800566 Polymorphism and Promoter Methylation With the Risk of Preeclampsia

**DOI:** 10.1155/omcl/1490896

**Published:** 2025-11-17

**Authors:** Maryam Pourmahmood, Somayeh Rahimi, Nayebali Rezvani, Ebrahim Shakiba, Zohreh Rahimi

**Affiliations:** ^1^Department of Clinical Biochemistry, Medical School, Kermanshah University of Medical Sciences, Kermanshah, Iran; ^2^Student Research Committee, Kermanshah University of Medical Sciences, Kermanshah, Iran; ^3^Medical Biology Research Center, Health Technology Institute, Kermanshah University of Medical Sciences, Kermanshah, Iran

**Keywords:** methylation, NQO1, oxidative stress, polymorphism, preeclampsia, qMSP

## Abstract

**Background:**

Oxidative stress plays a crucial role in the pathogenesis of preeclampsia. Given that the NADPH quinone oxidoreductase 1 (NQO1) is an important enzyme in the antioxidant system, this study aimed to investigate the relationship between the NQO1 rs1800566 polymorphism, NQO1 promoter methylation, and oxidative stress with the risk of preeclampsia.

**Methods:**

This case-control study analyzed 170 women, including preeclampsia patients and healthy pregnant women. To investigate the NQO1 rs1800566 variants, the polymerase chain reaction-restriction fragment length polymorphism (PCR-RFLP) method was used. Promoter methylation analysis in 96 of these samples was conducted using quantitative methylation-specific PCR (qMSP) method. Glutathione peroxidase (GPx) and superoxide dismutase (SOD) activity, along with zinc (Zn), copper (Cu), selenium (Se), malondialdehyde (MDA), and total antioxidant capacity (TAC) levels were measured using chemical methods.

**Results:**

We found reduced levels of TAC, Zn, and Se, and also the SOD activity in patients than controls. However, the MDA and Cu levels, and the GPx activity increased in preeclamptic patients. No association was identified between the NQO1 rs1800566 variants or NQO1 promoter methylation with the risk of preeclampsia.

**Conclusion:**

It seems the NQO1 rs1800566 and the promoter methylation of NQO1 gene are not involved in the risk of preeclampsia. However, our findings indicate the presence of oxidative stress in preeclamptic patients.

## 1. Introduction

Preeclampsia is a pregnancy-related disorder defined by the hypertension (systolic and diastolic pressure ≥140/90 mmHg) and proteinuria (≥300 mg/24 h) occurring after 20 weeks of gestation. Preeclampsia affects multiple organ that poses serious risks to both mother and fetus [1, 2]. This complication of pregnancy impacts 2%–15% of pregnancies, leading to over 4 million diagnoses annually and contributing to approximately 70,000 maternal and 500,000 neonatal deaths [3]. While the precise etiology remains unclear, it is understood that a complex interplay of genetics, epigenetics, and environmental factors contributes to its pathophysiology. In preeclampsia, impaired trophoblast invasion into the uterine spiral arteries is thought to cause poor placental perfusion, ischemia reperfusion injury, and the generation of reactive oxygen species (ROS), leading to oxidative stress [4, 5].

Various enzymes play critical roles in regulating the balance between ROS production and detoxification. One of these key enzymes is NAD(P)H quinone oxidoreductase 1 (NQO1), sometimes referred to as DT-diaphorase, that is encoded by the NQO1 gene on chromosome 16q22.1 [6]. NQO1 catalyzes the reduction of quinones to hydroquinones, preventing the formation of harmful semiquinone radicals, and thereby limiting ROS production. The enzyme requires a flavin adenine dinucleotide (FAD) cofactor for its functions [7]. NQO1 detoxifies harmful quinones, maintains P53, and is involved in cellular defense against oxidative stress and inflammation [8]. Additionally, oxidative stress triggers Nrf2 to regulate cellular redox balance by controlling more than 200 genes that are its downstream, including secondary response enzymes like heme oxygenase-1 (HO-1), glutathione S-transferases (GST), catalase (CAT), superoxide dismutase (SOD), and NQO1 [7, 9]. Several studies have demonstrated that this regulatory redox system is disrupted in preeclampsia [10, 11]. Moreover, the protective role of NQO1 has been shown in some diseases that are associated with high blood pressure [8, 12, 13], but its specific involvement in preeclampsia has not been well investigated.

A common polymorphism in the NQO1 gene, rs1800566 (C609T), reduces the enzyme affinity for FAD, thereby diminishing its antioxidant capacity. Several studies have reported significant associations between this polymorphism and increased susceptibility to oxidative stress-related disorders, including cardiovascular diseases [2], neurological disorders [14, 15],diabetic nephropathy [15], and various cancers [16–18]. However, other investigations have found no significant association, and in some populations, potential protective effects have also been observed, for example in hepatocellular, lung, kidney, or bladder cancers [19–22].

In addition to genetic variations, epigenetic modifications like DNA methylation, can significantly affect the regulation of genes. In preeclampsia, methylation patterns differ significantly from normal pregnancy affecting genes involved in developmental and immune responses [23]. Although it has been shown that the expression of the NQO1 gene in placenta tissue decreases in preeclampsia [24], no study has been conducted on the regulation of the gene expression and the methylation status of this gene. Meanwhile, it has been shown that NQO1 promoter methylation suppresses gene expression in various diseases such as some cancers [25, 26].

Therefore, this study aims to investigate the possible relationship between the NQO1 rs1800566 variants and NQO1 promoter methylation status in preeclampsia patients. Furthermore, we analyzed various oxidative and antioxidant markers, including malondialdehyde (MDA), SOD, glutathione peroxidase (Gpx), total antioxidant capacity (TAC), as well as trace elements such as zinc (Zn), selenium (Se), and copper (Cu), to evaluate the redox balance in patients with preeclampsia. Understanding this association could provide new viewpoints on the regulatory processes involved in the molecular pathogenesis of preeclampsia.

## 2. Materials and Methods

### 2.1. Characteristics of the Population and Specimen Processing

In the current case-control research, the preparation and the collection of samples were based on the previous study [27]. We examined the NQO1 rs1800566 polymorphism in 80 women with normal pregnancy and 90 preeclamptic women (including 59 cases with mild preeclampsia and 31 cases with severe preeclampsia). These participants were recruited from obstetric clinics in the Mazandaran Province of Iran between September 2018 and October 2019. Also, we examined the promoter methylation status of the NQO1 gene in these samples, focusing on 47 individuals with healthy pregnancy and 49 individuals diagnosed with preeclampsia.

The diagnostic criteria for preeclampsia included increased arterial blood pressure (systolic ≥140 mmHg and diastolic ≥90 mmHg, measured on two distinct instances at a minimum interval of 4 h after 20 weeks of pregnancy), proteinuria (≥300 mg in a 24-h urine sample and a urine protein-to-creatinine ratio ≥ 0.3), and dysfunction of other maternal organs, like acute kidney failure (serum creatinine level ≥1.1 mg/dL), liver dysfunction (increasing serum transaminase levels more than twice the normal limit or having epigastric pain), or intrauterine growth restriction of the fetus [28]. Patients with a blood pressure of ≥160/110 mmHg on two separate occasions, with proteinuria of ≥3+ in two random urine samples, or presenting symptoms like headache, epigastric pain, elevated liver enzymes or creatinine, hemolysis, pulmonary edema, or intrauterine growth restriction were classified as having severe preeclampsia [29]. The study excluded participants with diabetes mellitus, multiple pregnancies, and any autoimmune or chronic illnesses. The healthy control group consisted of pregnant women who had no complications or conditions associated with high blood pressure.

Data on demographics, clinical characteristics, and delivery details were gathered using questionnaires, in-person interviews, and medical records. A total of 6 mL of blood was obtained from each participant. Three milliliters were centrifuged without anticoagulants to isolate serum, which was then preserved at −80°C for biochemical evaluation. The other 3 mL were collected in EDTA tubes for DNA isolation and later genetic analysis.

The research received approval from the Ethics Committee of the Kermanshah University of Medical Sciences (approval ID: IR.KUMS.MED.REC.1401.281), and all participants gave written consent after being fully informed of the study.

### 2.2. Biochemical Assessment

The serum levels of Cu, Zn, and Se were quantified using a Graphite Furnace Atomic Absorption Spectrometer (Model PG 990, PG Instruments Ltd., China) according to established procedures [30, 31]. Also, the activities of SOD and GPx enzymes in the serum were assessed with prepackaged enzymatic test kits (Kiazist, Hamedan, Iran) as outlined by the kit provider's protocol.

MDA, a by-product of lipid peroxidation, is widely recognized as a marker for oxidative stress. Serum MDA levels were assessed using a commercially available kit (Teb Pazhouhan Razi, Tehran, Iran). In this method, MDA reacts with thiobarbituric acid (TBA) under acidic conditions and elevated temperatures to produce a mixture of MDA and TBA, which is then determined through colorimetric measurement. The amount of absorption at 540 nm was quantified utilizing a microplate reader (ELx808, BioTek, Winooski, VT, USA).

The serum TAC level was determined using a colorimetric assay kit (Kiazist, Hamedan, Iran). This method relies on the reduction of Cu^2+^ to Cu^+^ by serum antioxidants in the presence of a chromogen substance, forming a chromatic compound that was quantified by measuring absorbance at 450 nm.

### 2.3. NQO1 Polymorphism (rs1800566) Assessment

DNA was isolated from white blood cells through the phenol–chloroform method [32]. The amount of DNA was measured using the Nanodrop spectrophotometer (Thermo Scientific, USA). The polymerase chain reaction-restriction fragment length polymorphism (PCR-RFLP) method was used to determine the genotypic pattern. The PCR reaction's primer sequences for NQO1(rs1800566) were as the forward primer of 5′-AAG CCC AGA CCA ACT TCT-3′ and the reverse primer of 5′- ATT TGA ATT CGG GCG TCT GCT G -3′. The PCR reaction was carried out in a total volume of 25 µL, comprising 12.5 µL of master mix, 1 µL of each primer, 1 µL of template DNA, and 9.5 µL of nuclease-free water. An Eppendorf thermal cycler with the following thermal profile carried out the amplification process: an initial denaturation at 94°C for 5 min, followed by 35 cycles of 94°C for 30 s, 66°C for 30 s, and 72°C for 30 s. A final extension was carried out at 72°C for 5 min and finally a product of 174 bp was obtained. The quality of the PCR products was verified by electrophoresis on a 2% agarose gel. The PCR product was digested by the restriction enzyme HinfI (Thermo Fisher Scientific, USA) following a protocol that uses 15 µL of amplified PCR product, 3 µL of buffer R, 2 µL of nuclease-free water, and 0.2 µL of enzyme, incubated at 37°C for 24 h. To identify the target genotype, the RFLP products were put on a 3% agarose gel. The 174 bp PCR product remains intact in the presence of the CC genotype, whereas in the presence of the TT genotype the PCR product digested into 119- and 55 bp fragments. The CT genotype was identified on the gel by three distinct fragments of 174-, 119-, and 55-bp.

### 2.4. NQO1 Promoter Methylation Status Assessment via Quantitative Methylation-Specific PCR (qMSP)

We used the Methylation-Gold Kit from ZYMO Research (D5006, USA) for DNA treatment with sodium bisulfite to differentiate between methylated and unmethylated CpG sites. This process converted unmethylated cytosines in DNA to uracil.

The Eukaryotic Promoter Database (EPD) was used to determine the promoter region of the NQO1 gene [33]. The UCSC database (https://genome.ucsc.edu/) was used to extract the CpG island sequences [34]. Methylated and unmethylated primers were developed with the Methyl Primer Express software. The Oligoanalyzer program was used for analyzing secondary structures, and the primers were BLAST at NCBI to make sure they did not bind to genomic DNA (nonbisulfite). The sequences of methylated and unmethylated primers are listed in the Table 1. The qPCR reactions were performed using the qPCRBIO SybrGreen Mix Separate-ROX master mix based on the manufacturer's instructions and by using the StepOne Plus Real Time PCR platform (Applied Biosystems, USA). For the methylation reaction, the required volumes were 5 µL of qPCR master mix, 0.15 µL of forward primer, 0.3 µL of reverse primer, 0.15 µL of ROX, 1 µL of bisulfite-treated DNA, and 3.4 µL of DNase-free water. For the unmethylated reaction, the volumes included 5 µL of master mix, 0.3 µL of each primer, 0.15 µL of ROX, 1 µL of DNA, and 3.25 µL of nuclease-free water. For both methylated and unmethylated DNA, the PCR protocol began with an initial denaturation at 95°C for 2 min. The cycling phase consisted of three steps. The first step was conducted at 95°C for 15 s. For the methylation process, the annealing occurred at 54°C for 25 s, followed by the extension at 72°C for 25 s. For evaluation of unmethylation, the annealing step was performed at 60°C for 20 s, and the extension at 72°C for 20 s. At last, the characteristics of the products were confirmed by the melting curve analysis. The PCR amplicons were subsequently confirmed through electrophoresis on a 2.5% agarose gel to assess their specificity. Finally, the percentage of methylation was calculated by the following formula [35]:

  
$$\begin{array}{c} \text{Meth}\%=100/\left[1+2^{\Delta \text{Ct }\left(\text{meth}-\text{unmeth}\right)}\right]\;\%. \end{array}$$

### 2.5. Statistics Analysis

SPSS software version 16 was used for analysis. If the data distribution was expected normal, the independent *t*-test was used to compare the means; if not, the Mann–Whitney test was used. The one-way ANOVA test was utilized to compare quantitative data across several groups, while the chi-square test was used to compare qualitative variables. Also, the deviation genotype frequencies from the Hardy–Weinberg equilibrium (HWE) were calculated by chi-square test. Statistical significance was considered for *p*-values less than 0.05.

## 3. Results

Table 2 presents the demographic and clinical features of both patients and controls. Prepregnancy and pregnancy BMI were notably higher in patients with preeclampsia, including both severe and mild forms, than in healthy pregnant women. Newborn weight was significantly reduced in both severe and mild cases of preeclampsia compared to the healthy revise as group. Also, intrauterine growth retardation (IUGR) was present in 12.2% of newborns of preeclamptic mothers compared to the absence of IUGR in the newborns of healthy mothers (Table 2). In Table 3 the levels of antioxidants elements and enzymes and oxidative stress parameters in patients and controls are depicted. There were significantly lower Zn, Se, and TAC levels, along with reduced SOD enzyme activity, in all preeclamptic patients, including those with mild and severe preeclampsia, compared to the reference group. However, Cu level, GPx activity, and the MDA level were significantly elevated in all patients, including those with mild and severe preeclampsia compare to the control group (Table 3).

In this study, the distribution of NQO1 rs1800566 genotypes was in HWE (*χ*^2^ = 2.13, *p* > 0.1). The distribution of NOQ1 genetic variants and allelic frequencies among patients and the control group is presented in Table 4. No significant differences were detected in the frequencies of NOQ1 genotypes between patients and controls. The frequency of NQQ1 T allele was 22.8% in all preeclamptic patients that was not significantly different compared to controls (21.2%, *p* = 0.73) (Table 4).

The levels of antioxidant enzymes, and the parameters of oxidative stress were compared between various genotypes of NQO1 in all studied individuals. No notable variations were detected in the levels of biochemical indicators between three genotypes of NQO1 (Table 5). The NQO1 methylation status in cases and the control group is presented in Table 6. The mean methylation rate of NQO1 was 44.75 ± 35.80% in all patients with preeclampsia compared to 45.50 ± 31.21% (*p* = 0.91) in controls (Table 6).

## 4. Discussion

Preeclampsia results from insufficient trophoblast invasion, which causes repeated hypoxia-reoxygenation cycles and oxidative stress. This process disrupts trophoblast function, triggering inflammation and advancing the disease [5]. Oxidative stress is a critical factor in the pathogenesis of preeclampsia, characterized by an imbalance between ROS production and antioxidant defense [36]. NQO1 is an enzyme that defends cell membranes against oxidative damage by reducing quinones [37]. Reduced expression of NQO1 in preeclampsia may lead to heightened susceptibility to lipid peroxidation [24]. Although genetic variants of NQO1 were not evaluated in their study, this finding raise the question of whether polymorphisms may contribute to this alteration. The NQO1 gene is known to have multiple variants, with at least five significant polymorphisms identified, including the extensively studied rs1800566 or C609T (Pro187Ser) [38]. The NQO1 rs1800566 polymorphism is associated with the decreased enzymatic activity; individuals homozygous for the T allele exhibit only 2%–4% of the wild-type enzyme activity, while heterozygotes show a threefold reduction compared to wild-type carriers [18]. The T allele of this variant is linked to several diseases [2, 39]. However, in some cases, the C allele may be a risk factor [15]. Previous studies have reported conflicting results regarding the variants of this polymorphism. In some cases, it was reported no association with the disease risk, while in others, it was linked only to specific populations [40, 41]. The conflicting findings regarding this polymorphism across different studies may be attributed to the ethnic differences, as well as variations in disease type and severity. Our study demonstrated the absence of correlation between NQO1 rs1800566 and preeclampsia among Iranian women. Furthermore, there was no association between the variants of the NQO1 polymorphism and susceptibility to mild and severe preeclampsia. Similarly, Zhao L. et al. [42] study in a Chinese population reported that this genetic variation was unrelated to the risk of preeclampsia, aligning with our findings.

In the current investigation we found the existence of oxidative stress in preeclamptic patients compared to controls reflected in the imbalance between body oxidant/antioxidant systems toward higher levels of oxidants. Our research showed that in preeclamptic patients, the levels of TAC, Zn, Se, and the SOD activity decreased, whereas the levels of MDA, Cu, and the GPx activity elevated. Cu and Zn are crucial components of the SOD, while Se is an essential element of the GPx. These two enzymes and their required elements are crucial for antioxidant defense [43]. Changes in serum trace element levels during pregnancy are linked to hypertensive disorders in pregnant mothers [44]. Our findings on trace elements are generally consistent with previous meta-analysis studies. They indicate that preeclampsia is associated with lower Zn and Se levels [45–47] and higher Cu levels in most populations, although these meta-analyses also reported regional and methodological heterogeneity [48, 49]. Consistent with our study, most investigations have demonstrated that MDA levels are elevated while TAC levels and SOD activity are reduced in preeclampsia. The divergent alteration of these parameters clearly reflects the disturbed redox balance in preeclampsia. SOD is a key antioxidant enzyme responsible for the removal of superoxide anions, and its depletion occurs due to counteraction increased oxidant levels in the cell. GPx is an NADPH-dependent enzyme that regenerates reduced glutathione (GSH) from its oxidized form (GSSG), thereby sustaining the intracellular GSH pool for antioxidant defense. Regarding GPx alteration in preeclampsia, there is no consensus. Some studies, consistent with our study, it was increased and considered a compensatory effect against increased oxidative stress, while in others, it was shown to be decreased [45–49]. Such discrepancies in antioxidant system parameters across studies may stem from variations in the study design, sample size, genetics and epigenetics factors, or the characteristics of the studied population. Furthermore, factors like the age of gestation during sampling and the specific preeclampsia subtypes examined could affect the results. Furthermore, we did not observe a significant difference between the levels of oxidative stress biomarkers according to the NQO1 genotypes.

To investigate the promoter methylation status of NQO1 in the blood of pregnant women, we performed a qMSP analysis. DNA methylation could impact gene expression by adding methyl groups to CpG-rich sites, especially in the gene promoter, thereby preventing transcription factors from binding. Researches have indicated that methylation alterations in NQO1 can impact its expression in various diseases [26, 50–52]. Ding et al. [24] found that the NQO1 expression decreased in placental tissue in preeclampsia patients. However, the promoter methylation of this gene has not yet been investigated in preeclampsia. While the placenta is the most relevant organ in the pathogenesis of preeclampsia, its collection is invasive and usually possible after delivery. This limits its utility for biomarker discovery and early disease assessment. Therefore, our study focused on analyzing the methylation status of NQO1 in blood samples. Our study is the first to analyze the methylation status of the NQO1 gene in preeclamptic patients in comparison to unaffected pregnant women. Our results indicated the lack of a significant difference in the methylation status of NQO1 promoter between patients and controls. Furthermore, there was no variation in the gene methylation associated with the disease severity. As the methylation pattern of this gene might be tissue-specific, determination of methylation status of the gene in placenta tissue, which plays a central role in preeclampsia, may provide further insights about its molecular mechanism. It is also important to investigate the other epigenetic modifications to assess the expression regulation of NQO1 in preeclampsia.

## 5. Conclusion

The present study observed the absence of a link between the NQO1 rs1800566 variants and susceptibility to preeclampsia and the lack of an association between NQO1 methylation status with the risk of preeclampsia. Also, our study revealed the presence of oxidative stress in preeclamptic patients. This study is the first one to investigate the NQO1 gene polymorphism and the promoter methylation status among Iranian patients with preeclampsia. Nevertheless, further research with larger sample sizes among various populations could help to elucidate the role of NQO1 gene variants and the NQO1 methylation in preeclampsia.

## Figures and Tables

**Table 1:** The sequences of methylated and unmethylated primers.

Methylated primers	
Forward	5′-TAGGATTTAGGCGTTGGGTTTC-3′
Reverse	5′-CTCGATAAACTAAACGACTCCG-3′

**Unmethylated primers**	

Forward	5′-AGGATTTAGGTGTTGGGTTTTG-3′
Reverse	5′-CTCTCAATAAACTAAACAACTCCA-3′

**Table 2:** Characteristics of preeclamptic patients and healthy pregnant women.

Parameters	All preeclamptic patients *n* = 90 mean ± SD (*p*)	Severe preeclampsia *n* = 31 mean ± SD (*p*)	Mild preeclampsia *n* = 59 mean ± SD (*p*)	Controls *n* = 80 mean ± SD (*p*)
Maternal age (year)	31.3 ± 5.5 (<0.001)	32.1 ± 5.9 (<0.001)	30.7 ± 5.3 (<0.001)	26.2 ± 5.9

Gestational age (weeks)	35.0 ± 3.9 (<0.001)	34.5 ± 3.7 (<0.001)	35.4 ± 3.9 (<0.001)	39 ± 1.1

Before pregnancy weight (kg)	76.2 ± 16.2 (<0.001)	71.5 ± 16.2 (0.14)	78.3 ± 15.6 (<0.001)	65.7 ± 12.7

After pregnancy weight (kg)	89.4 ± 17.7 (<0.001)	84.5 ± 16.7 (0.044)	91.6 ± 17.8 (<0.001)	76.5 ± 13.5

Before pregnancy BMI (kg/m^2^)	29.0 ± 5.7 (<0.001)	27.6 ± 6.1 (0.15)	29.7 ± 5.4 (<0.001)	25.5 ± 4.6

After pregnancy BMI (kg/m^2^)	34.1 ± 6.3 (<0.001)	32.6 ± 6.3 (0.043)	34.8 ± 6.3 (<0.001)	29.7 ± 4.9

Systolic blood pressure (mm Hg)	149.3 ± 13.6 (<0.001)	158.7 ± 16.3 (<0.001)	144.0 ± 8.0 (<0.001)	107.3 ± 17.2

Diastolic blood pressure (mm Hg)	93.2 ± 8.8 (<0.001)	98.7 ± 10.9 (<0.001)	90.1 ± 5.6 (<0.001)	71.3 ± 7.0

Newborn weight (g)	2548.8 ± 836.1 (<0.001)	2174.8 ± 967.9 (<0.001)	2770.9 ± 729.3 (<0.001)	3364 ± 412.9

IUGR No Present	79 (87.8%) 11 (12.2%) (<0.001)	22 (71%) 9 (29%) (<0.001)	57 (96.6%) 2 (3,4%) (0.097)	80 (100%) 0

Proteinuria No +1 +2 +3 +4	2 (2.2%) 57 (63.3%) 14 (15.6%) 14 (15.6%) 3 (3.3%) All *p* < 0.001	2 (6.5%) 12 (38.7%) 4 (12.9%) 11 (35.5%) 2 (6.5%) All *p* < 0.001	0 (0.0%) 45 (76.3%) 10 (16.9%) 3 (5.1%) 1 (1.7%) All *p* < 0.001	78 (97.5%) 2 (2.5%) 0 0 0

Family history	7 (7.8%) (0.011)	3 (9.7%) (0.005)	4 (6.8%) (0.018)	0

Abbreviations: BMI, body mass index; IUGR, intrauterine growth retardation.

**Table 3:** The levels of antioxidants elements and enzymes and oxidative stress parameters in patients and controls.

Parameters	All preeclamptic patients *n* = 150 Mean ± SD (*p*)	Severe preeclampsia *n* = 31 Mean ± SD (*p*)	Mild preeclampsia *n* = 59 Mean ± SD (*p*)	Controls *n* = 80 Mean ± SD
Zn (μg/dL)	39.6 ± 16.1 (<0.001)	27.5 ± 8.0 (<0.001)	46.3 ± 15.6 (<0.001)	68.4 ± 18.1
Cu (mg/L)	1.81 ± 0.51 (<0.001)	1.79 ± 0.57 (<0.003)	1.83 ± 0.48 (<0.001)	1.49 ± 0.33
Se (μg/L)	54.3 ± 18.4 (<0.001)	48.4 ± 16.4 (<0.001)	57.3 ± 19 ( < 0.001)	71.7 ± 22.9
SOD (U/mL)	5.63 ± 2.41 (<0.001)	4.3 ± 1.7 (<0.001)	6.3 ± 2.5 (<0.001)	9.0 ± 2.4
GPx (mU/mL)	6.72 ± 2.23 (0.001)	6.99 ± 2.34 (0.006)	6.54 ± 2.16 (0.036)	5.58 ± 1.70
Serum TAC (mmol/L)	3.36 ± 1.21 (<0.001)	3.2 ± 0.72 (<0.001)	3.45 ± 1.41 (<0.001)	4.45 ± 0.99
Serum MDA (µM)	4.33 ± 0.72 (<0.001)	4.97 ± 0.50 (<0.001)	3.98 ± 0.58 (<0.001)	3.47 ± 0.44

**Table 4:** Comparison of genotype and allele frequencies of NQO1 between patients and controls.

Genotypes	All preeclamptic patients *n* = 90 *n* (%)	Severe preeclampsia *n* = 31 *n* (%)	Mild preeclampsia *n* = 59 *n* (%)	Controls *n* = 80 *n* (%)
NQO1 C >T				
CC	51 (56.7)	18 (58.1)	33 (55.9)	49 (61.2)
CT	37 (41.1)	12 (38.7)	25 (42.4)	28 (35)
TT	2 (2.2%)	1 (3.2)	1 (1.7%)	3 (3.8)

	*χ* ^2^ = 0.9, *p* = 0.63	*χ* ^2^ = 0.14, *p* = 0.93	*χ* ^2^ = 1.14, *p* = 0.56	

Alleles C	139 (77.2)	48 (77.4)	91 (77.1)	126 (78.8)

T	41 (22.8) *χ*^2^ = 0.11, *p* = 0.73	14 (22.6%) *χ*^2^ = 0.047, *p* = 0.82	27 (22.9) *χ*^2^ = 0.1, *p* = 0.74	34 (21.2)

**Table 5:** Biochemical parameters according to the NQO1 genotypes in all studied individuals.

NQO1				
Parameters	CC *N* = 100	CT *N* = 65	TT *n* = 5	*p*
Zn (μg/dL)	54.3 ± 23.4	52.4 ± 21.1	41.8 ± 12.2	0.44
Se (μg/L)	61.0 ± 21.9	65.2 ± 23	56.0 ± 22.6	0.40
Cu (mg/L)	1.68 ± 0.44	1.61 ± 0.49	1.73 ± 0.43	0.57
SOD (U/mL)	7.54 ± 3.10	6.77 ± 2.67	6.11 ± 1.1	0.18
GPx (mU/mL)	6.0 ± 2.1	6.5 ± 2.0	6.98 ± 1.31	0.24
TAC (mmol/L)	3.77 ± 1.19	4.1 ± 1.28	3.22 ± 1.25	0.12
MDA (µM)	3.9 ± 0.73	3.96 ± 0.75	4.0 ± 0.83	0.87

**Table 6:** NQO1 methylation status in patients and controls.

Methylation	All preeclamptic patients *n* = 49	Severe preeclampsia *n* = 19	Mild preeclampsia *n* = 30	Controls *n* = 47
Overall methylation percent	44.75 ± 35.80 *p* = 0.91	41.70 ± 34.5 *p* = 0.90	46.70 ± 37.0 *p* = 0.98	45.50 ± 31.21

Methylation lower than 50%	*n* = 27, 55.1%	*n* = 12, 63.2%	*n* = 15, 50.0%	*n* = 26, 55.3%

Methylation higher than 50%	*n* = 22, 44.9% *χ*^2^ = 0.0, *p* = 0.98	*n* = 7, 36.8% *χ*^2^ = 0.34, *p* = 0.56	*n* = 15, 50.0% *χ*^2^ = 0.2, *p* = 0.64	*n* = 21, 44.7%

## Data Availability

The data that support the findings of this study are available from the corresponding author upon reasonable request.
